# Tandem Domains with Tuned Interactions Are a Powerful Biological Design Principle

**DOI:** 10.1371/journal.pbio.1002306

**Published:** 2015-11-30

**Authors:** Ruth Nussinov, Chung-Jung Tsai

**Affiliations:** 1 Cancer and Inflammation Program, Leidos Biomedical Research, Inc., Frederick, Maryland, United States of America; 2 Frederick National Laboratory for Cancer Research, National Cancer Institute, Frederick, Maryland, United States of America; 3 Sackler Institute of Molecular Medicine, Department of Human Genetics and Molecular Medicine, Sackler School of Medicine, Tel Aviv University, Tel Aviv, Israel

## Abstract

Allosteric effects of mutations, ligand binding, or post-translational modifications on protein function occur through changes to the protein’s shape, or conformation. In a cell, there are many copies of the same protein, all experiencing these perturbations in a dynamic fashion and fluctuating through different conformations and activity states. According to the “conformational selection and population shift” theory, ligand binding selects a particular conformation. This perturbs the ensemble and induces a population shift. In a new *PLOS Biology* paper, Melacini and colleagues describe a novel model of protein regulation, the “Double-Conformational Selection Model”, which demonstrates how two tandem ligand-binding domains interact to regulate protein function. Here we explain how tandem domains with tuned interactions—but not single domains—can provide a blueprint for sensitive activation sensors within a narrow window of ligand concentration, thereby promoting signaling control.

## Abstract 2

What is the evolutionary advantage of a tandem domain organization rather than a single domain? Based on new observations of the regulation of protein kinase A by cAMP, this Primer explains how double-conformational selection can enable highly sensitive signaling sensor performance.

Classical biology considers organisms, tissues, cells, and molecules. It views molecules as objects that may associate or dissociate and molecular mechanisms as series of events turning molecules on or off. Such traditional views are often captured by pathway diagrams, where molecules are depicted by some simple geometrical shapes (circles, boxes) and their subsequent interactions (activation, inhibition) by arrows or bar-capped edges [1,2]. Such descriptions are powerful since they provide an overall picture and thus help in experimental design; however, they do not allow detailed mechanistic understanding. They are not able to explain how oncogenic mutations could activate the protein, making it signal even in the absence of an incoming cue, or why a post-translational modification (PTM) away from the binding site is essential for function. The absence of mechanistic detail hampers molecular design, which aims to tune regulation of cellular signaling. Structures are not rocks; and the only way to gain insight is to consider molecules as ensembles of multiple interconverting conformations, where the relative population of a certain conformation, or “state”, is dynamic [3]. Ensembles are not fixed; they fluctuate, and their fluctuations reflect changes in the physical environment, such as mutations, PTMs, or ligand binding. Binding events take place through “conformational selection”; the outcome is a “population shift” [4].

How can conformational selection lead to a steep activation of a protein within a narrow range of ligand concentration? The regulation of protein kinase A (PKA) by its regulatory subunit (RIα) suggests that evolution has come up with an elegant solution: the engineered RIα homologous tandem cAMP binding domains (CBD-A, CBD-B) organization. When not bound to cAMP, RIα is in an open conformation, with weak interactions between the domains. With higher affinity, CBD-B is the first to bind cAMP. As Melacini and his colleagues show [5], binding shifts the RIα ensemble. This has two consequences: it increases the population of a cAMP-compatible conformation in CBD-A, which now also binds cAMP, and in particular strengthens the weak CBD-A/CBD-B domain–domain interactions. Even though the cAMP-elicited conformational change in each of the domains is minor, the global conformational change of RIα from an open to a closed conformation is large. This is important since the stable closed RIα conformation has steric conflicts with PKA. The more stable closed conformation now shifts the equilibrium away from the open state, and the freed PKA is activated. Note that even in the absence of cAMP, holo-like closed RIα conformations are present in the ensemble; however, the populations of these states are functionally insignificant. Why is such a mechanism advantageous to the cell? It allows PKA an efficient cAMP concentration-sensitive molecular response. Protein design efforts aiming at efficient activation through sharp concentration transitions may consider exploiting such a strategy. Fig 1 illustrates how this mechanism works.

**Fig 1:**
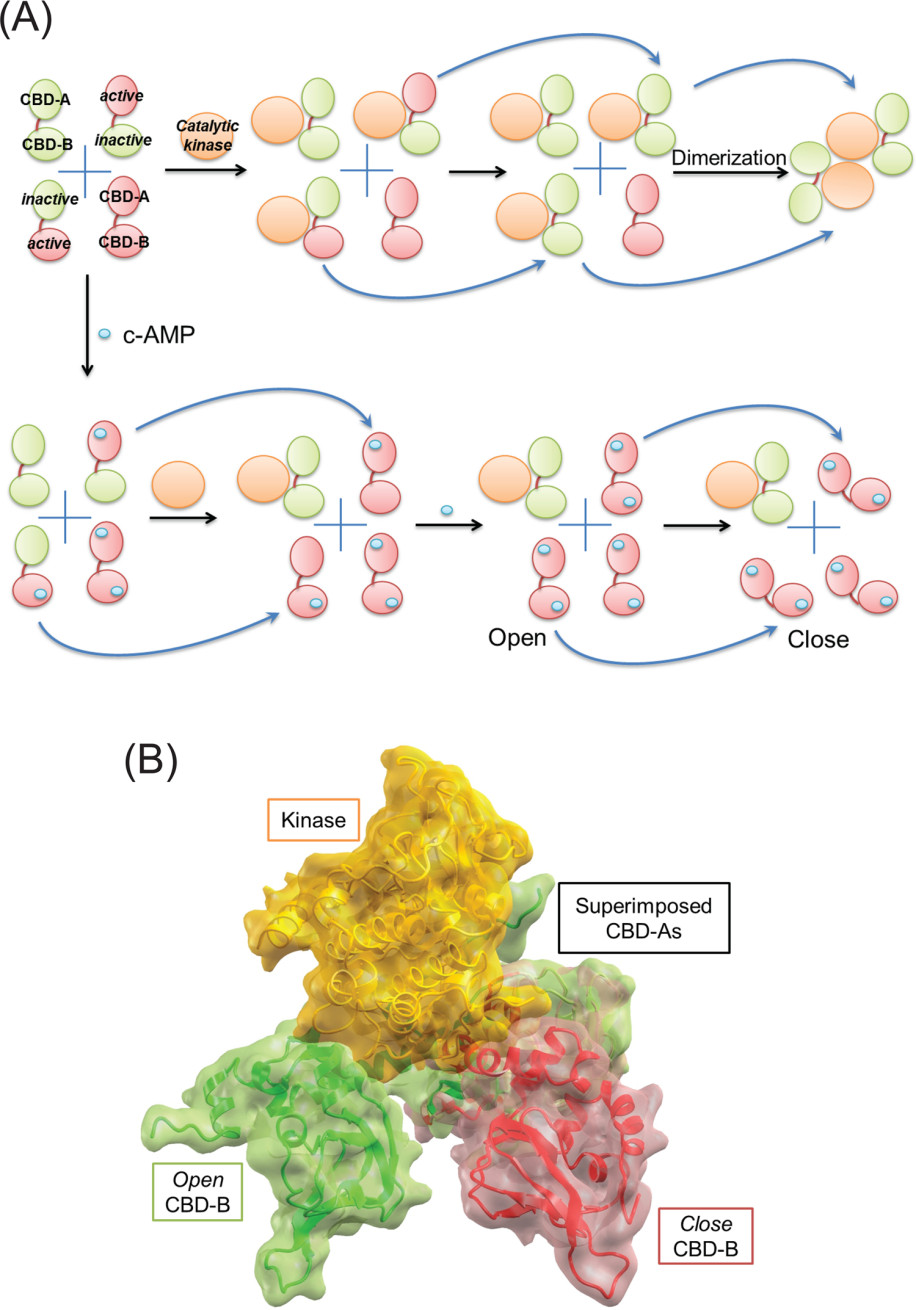
The role of conformational selection and population shift in regulation of PKA activity. (A) We start with four independent population states of PKA regulatory subunit (RIα) with two tandem cAMP binding domains (CBD-A and CBD-B) either in active (small red oval) or inactive (small green oval) states, presumably in open arrangements that have been verified by the NMR (nuclear magnetic resonance) experiments [5]. The top panel shows how the catalytic subunit of PKA (large orange oval) is secured in the inhibited (OFF, inactive) state by the regulatory subunit in the absence of cAMP. First, through conformation selection, the kinase domain only binds to inactive CBDs. The two tandem CBDs can exist in three possible populations (inactive–inactive, active–inactive, and inactive–active), with different binding affinities between them. Then, thanks to the tandem arrangement of the CBDs and/or via the allosteric influence resulting from the inactive CBD binding, the other, active CBD states are further stabilized in the inactive form when bound to the kinase subunit as indicated by the blue curved arrows. To ensure limited activity in the absence of cAMP, the formation of a tetramer by dimerization (indicated by the arrows) further effectively reduces the free active kinase subunit. The bottom panel shows how cAMP (tiny cyan oval) maximizes the concentration of free catalytic kinase through its binding to the PKA regulatory subunit. First, the panel shows that cAMP selectively binds to inactive CBDs only. Then, the catalytic subunit is only permitted to bind to the population of the regulatory subunit with both inactive CBDs. Next, the cAMP-bound active CBD allosterically shifts the other, inactive CBD into active conformation (indicated by the arrows), which allows cAMP to occupy both binding sites in CBD. (B) Large (inter-CBDs) and small (intra-CBD) conformational changes can be seen through the superposition of CBD-A domains taken from the open tandem CBDs (green) bound to the catalytic kinase subunit (yellow) (PDB: 1 rgs) and the closed form of the regulatory subunit (red) (PDB: 2 qcs). The steric collision between the catalytic kinase subunit and the closed CBD-B domain seen in the figure reveals why the large conformational change further destabilizes substantially the binding affinity between CBD-A and the catalytic subunit, which has already been reduced by the small intra-CBD conformational change due to cAMP binding.

## Population Shift in PKA-RIα Leads to Sharp Activation

In the absence of cAMP, the PKA-RIα region spanning the tandem CBD-A and CBD-B domains (RAB) samples four states in the ensemble (Fig 1). Each CBD accesses two states: an active conformation that is complementary to cAMP and an inactive conformation, which is not. The inactive conformation can associate with PKA’s catalytic subunit (C), thereby deactivating it. The active RAB conformation is C-subunit binding incompetent. In the absence of cAMP, the populations of the two states in each of the domains are comparable. The RAB state with both domains in the inactive conformation has the highest affinity to the C-subunit. Because in the absence of cAMP there are no significant interactions between the two domains, the sampling of the two states in each of the domains is independent of each other.

**Fig 2:**
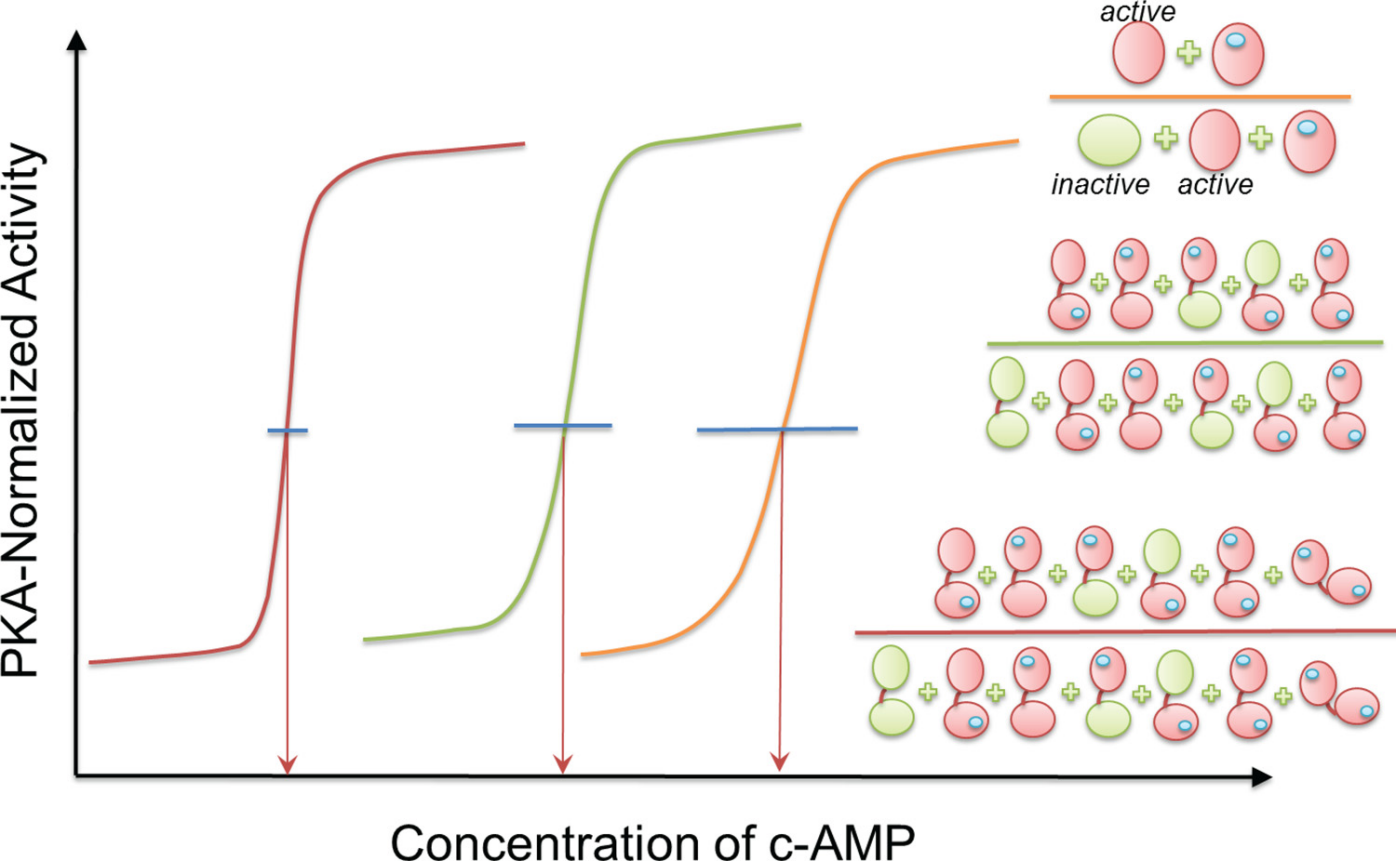
How the tandem CBDs in the PKA regulatory subunit adjust PKA’s activity as a cellular switch through the binding of second messenger cAMP. An ideal biological switch, here in the case of PKA activity as a function of cAMP concentration, is to establish a lower transition concentration (indicated by the red arrows at a middle point of maximum and minimum activity of PKA) and a narrow transition window (indicated by the length of blue horizontal lines) that passes the transition point and ends without a significant change of PKA activity with respect to cAMP concentration change. The orange transition curve on the right corresponds to a scenario where PKA regulatory subunit has only a single CBD. Both green and red transition curves are for PKA regulatory subunit with tandem CBDs; but the green curve does not have a significant population of the closed form with cAMP-bound CBDs (illustrated in Fig 1), as in the case of the W260 mutant. In the scenario depicted by the orange curve with a single CBD, the activity of PKA is proportional to [CBD^active^] + [CBD(cAMP)] / [CBD^inactive^] + [CBD^active^] + [CBD(cAMP)] if we assume that cAMP-bound CBD dominates the active conformation. The corresponding graphic presentation is given on the top right with orange divider line with individual species illustrated in Fig 1. In the scenario referred to by the green curve, PKA activity is proportional to the middle graphic presentation with the green divider. However, with the closed form, PKA activity shown by the red curve corresponds to the bottom graphic presentation with red divider. Note that this is only a schematic figure for clarity and only representative states are shown in the cartoons in the right side of the figure. As in the case of the tandem domains only the inactive–inactive state is explicitly shown for the apo form. For a full enumeration of states, see Fig 1A.

As shown by Fig 1, binding of cAMP changes the picture. Selected primarily by an active conformation of CBD-B—since the affinity of CBD-B to cAMP is higher than that of CBD-A—the binding shifts the RAB equilibrium. Even though binding to CBD-A can already lower the constant for the cAMP-dependent activation (Ka) of PKA (Fig 2), the binding of the CBD-B and association of the two domains explains the cAMP-dependent activation of PKA by more than an order of magnitude from 1 μM to 80 nM [6].

## Efficient Activation with Sharp Concentration Transition

Activation of PKA takes place via three steps [5]: first, selection of cAMP by an active conformation of CBD-B; second, selection of active conformation of CBD-A; and third, stabilization of interdomain interactions that are incompatible with the RIα:C interface. Fig 2 provides a schematic diagram illustrating the contributions of these steps to PKA activation with respect to cAMP concentration. With low affinity to the catalytic domain, cAMP-bound CBD-A can already activate PKA, albeit only at high concentrations of cAMP. Even though when not bound to cAMP the affinity of the inactive conformation of CBD-B to the C-subunit is lower than that of CBD-A, it is nonetheless sufficient to shift the RAB equilibrium toward the inactive state; however, when CBD-B is bound to cAMP, the affinity of RAB to the C-subunit decreases further. Stabilization of the closed conformation that sterically blocks interaction with the C-subunit results in a sharper concentration transition curve.

## The Principle: The Efficacy of Activation Is Determined by the Extent of Population Shift

How does an allosteric effector (in this case cAMP) drive a population shift such that in the active unbound state the two domains sample space independently of each other, which is not the case following binding of the effector? The most important principle is that the populations of the active and inactive state are determined by the free energy differences between them [7,8]. A specific function is decided by the extent that a macromolecule populates its active (or inactive) conformation. This implies that the efficacy of an allosteric effector is determined by the extent of the population shift. It is not determined by the binding affinity of the allosteric effector to the host allosteric pocket, or by the communication pathway between the allosteric and functional sites, although the pathway defines the residues that are important for the population shift. Second, ligand atoms can be divided into “anchor” and “driver”. Anchor atoms dock into the allosteric pocket, form favorable interactions, and stabilize the bound state. Driver atoms “pull” and/or “push” the receptor atoms, actions that shift the receptor population from the inactive to the specific active state (or vice versa). An attractive “pulling” or repulsive “pushing” by driver atoms can stabilize the active conformation and/or destabilize the inactive conformation. Driver atoms are responsible for the allosteric efficacy and anchor atoms for affinity, i.e., potency. Structural analysis of the active cAMP-bound “B” and inactive C-subunit-bound “H” crystal structures [9,10] pointed to the equatorial oxygen of the cyclic phosphate in cAMP, which is replaced by sulfur in antagonist R_p_-cAMP as the driver atom (ligand–host complexes, cAMP–R_A_ [PDB 3pna], and R_p_-cAMP–R_A_ [PDB 3plq]) [8].

## Why Nature Evolved a Multiple Conformational Selection Mechanism

The curves in Fig 2 highlight enzyme activity with each gained interaction. They emphasize how high activity can be reached efficiently through a narrow window of ligand concentration change. This could be of particular importance to an abundant enzyme with multiple critical functions in the cell, which is regulated by a second messenger, as is the case with PKA and cAMP. Nonetheless, even though the advantages are clear, it still remains to be seen how often this mechanism has been exploited by evolution. To date, most—if not all—mechanisms adopted by evolution were made use of in more than one system. Here nature has fine-tuned population shift for efficient tight functional control through concentration of a second messenger. Though challenging, design can follow these principles. Prediction of the transition concentration is critical for a successful design. The mechanistic details of how nature achieved efficient activation by tuning population shift explained here may provide a blueprint helping in encoding molecular programs that may yield desired system behaviors. Adjusting enzyme activity, which controls cellular switches, through a low effector transition concentration as we showed here for the case of PKA may establish useful—albeit challenging—guidelines. Tandem domains with finely tuned interactions can be a powerful design principle for sensitive functional control.

Thus, instead of forming tetramers to secure an inactive catalytic subunit, the fine tuning of the interdomain interactions between two inactive cAMP binding domains allosterically transforms the open structure of the tandem domains into the closed form. This further increases the total species of the regulatory unit that lose their abilities to bind and inhibit the PKA catalytic kinase. In this way nature achieves a critical regulation aim: binding of a ligand (cAMP) within a narrow concentration window can fully activate an enzyme (PKA) by releasing its catalytic subunit. The question is—can we, as a community, emulate it?

## Abbreviations

CBD-A: cAMP binding domain A
CBD-B: cAMP binding domain B
NMR: nuclear magnetic resonance
OFF: inactive; PKA, protein kinase A
PTM: post-translational modification
RAB: region spanning the tandem CBD-A and SB-B domains
RIα: PKA regulatory subunit
